# Effect of frame foundation geometry on the dynamic response of high-speed turbo machine foundations

**DOI:** 10.1016/j.heliyon.2024.e41050

**Published:** 2024-12-15

**Authors:** Ameer A. Ahmed, Mohammed Y. Fattah, Makki K. Mohsen

**Affiliations:** Civil Engineering Department, University of Technology, Baghdad, Iraq

**Keywords:** Ansys workbench, Frame foundation, Harmonic analysis, Modal analysis

## Abstract

The foundation of the turbo-generator comprises a generator, turbine, and associated equipment situated on the top of the frame foundation, which consists of a bottom raft, top deck, and columns. The current study examines how the geometrical characteristics of the frame foundation, such as slab thickness, column dimensions, and length, affect the dynamic displacement response and the natural frequencies of the frame foundation. The work is done through 3-D dynamic modal and harmonic analyses using Ansys Workbench finite element software. The impact of the top deck thickness was investigated using five thicknesses of (1.0, 1.2, 1.4, 1.6 and 1.8) m. To inspect the effect of the column's dimensions, the column's length was fixed for all cases, other frame dimensions are based on the prototype dimensions. The columns were assumed to be square with dimensions of (0.8, 0.9, 1.0, 1.1 and 1.2) m. The prototype slab, raft, and column dimensions are adopted for all cases to inspect the effect of the column's length. The column lengths of (6.8, 7.8, 8.8, 9.8, and 10.8) m were used to examine the column length effect on the frame foundation.

The study's results revealed that decreasing column length or increasing column dimensions increases the frame foundation's natural frequencies, and the frame's displacement response decreases remarkably. The results also revealed that changing the tabletop slab thickness has a marginal effect on the frame's dynamic performance. The results show that the behavior for different geometrical frame foundations can be presented by a single normalized curve for frame foundation.

## 1 Introduction

Considering static design principles, it is crucial to design machine foundations to ensure that all dynamic forces generated by machines are efficiently transferred to the soil through the foundation to eliminate any potential adverse consequence or deferential effects.

High-speed turbo-machines are one of the machine types that are used in various engineering applications including electrical plants, petrochemicals, refineries, and nuclear stations including steam turbines, gas turbines, other expanders, turbopumps, compressors, fans, motors, and centrifuges. These rotor machines are known for their high speed (3000–10000) rpm and their high cost. The cost of their foundation is a fraction of the machine's cost and hence the performance of the foundation is dominant for machine safety during their operation lifetime.

The foundations for steam turbine-generators, commonly referred to as turbine pedestals due to their substantial size, must be accurately engineered to withstand a range of load scenarios. These scenarios include stringent stiffness and vibration criteria mandated by both machine manufacturers and industrial standards, all while enduring cyclic and dynamically unbalanced forces. In practical terms, structural engineers rely on the foundation requirements outlined by the machine manufacturer and follow the applicable codes and standards during the design process. However, it's worth noting that manufacturer requirements can exhibit variations, and the application of codes or standards to such foundations often involves interpretation. As a result, many professionals in the field have developed their own distinct "proprietary" or "customized" methodologies, drawing from their personal experiences and preferences. It's important to recognize that some of these approaches may differ, and in some cases, even conflict with one another [1].

Many types of foundations are used for rotary machine types like block and frame foundations. Frame foundation is one type of foundation used for rotary machines; the machine is supported on the top slab. This slab in turn is maintained by the base raft and columns and the base raft rests on a group of piles or directly over the soil [2]. Block foundations are designed as rigid foundations while the frame foundations are considered to be flexible. In this situation, the machine is considered a non-elastic inertia body. In contrast, the tabletop and columns are considered as elastic inertia bodies, and soil is considered as elastic media. The dynamic behavior of a frame foundation is complex compared to that of a block foundation but the ability to make maintenance to the machine is superior for frame type foundation. Frame foundation is called an under-tuned foundation or low-tuned foundation because its vertical natural frequency is well below the machine's operating frequency. These types undergo transient resonance during starting and shutdown of the machine.

Mahesh and Irfan (2017) [3] conducted a comprehensive analysis of a Turbo-Generator machine foundation. Their approach involved modeling the foundation using Staad Pro software and subsequently conducting dynamic and static analyses. The Turbo-Generator itself was also subject to analysis and design through an analytical solution method. They opted to represent the superstructure as a space frame, while columns and beams were considered beam elements with six degrees of freedom. Dynamic analysis of the model was carried out to calculate the natural frequencies of the foundation in all three directions, as well as to assess the maximum vibration levels in both vertical and horizontal directions. Additionally, a static analysis was performed on the same model, and the design of the top deck beams and columns was completed based on the results of this analysis. The study involved calculating the natural frequencies and amplitude of vibration of the foundation in all three directions, with a comparison to the operating frequency. Ultimately, the design of the top deck beams was conducted by IS 2974 (part 3):1992, and it was established that the dimensions of the beams met the necessary criteria.

Abbas et al. (2017) [4], conducted a comprehensive study in which they used ABAQUS (v 6.13), finite element analysis, to inspect a block-type turbine-generator foundation's dynamic response situated at Al-Mansurya station in Iraq. Their analysis focused on the foundation's behavior under vertical harmonic excitation and utilized a layered-soil model with linear elastic properties. Importantly, the study considered both scenarios: one accounting for soil-structure interaction and another where this effect was disregarded. The soil-structure interaction was rigorously examined using a direct method, wherein the soil and structure were jointly analyzed in a single step. In addition to this, the researchers conducted Modal analysis to identify the natural frequencies and their mode shapes. Harmonic analysis was carried out as a vital component of their investigation. Ultimately, the study's findings underscored the critical importance of considering soil-structure interaction when analyzing such sensitive structures. It was firmly concluded that this interaction had a significant and noteworthy impact on the overall response of the foundation.

Sungyani and Desai (2017) [5], developed a computational model using SAP 2000 to examine the impact of the Kathmandu earthquake in 2015 on a tabletop foundation for turbomachinery. This analysis was conducted across five different soil conditions: generic soil, generic rock, very hard generic rock, NEHRP D class, and NEHRP C class. The study's findings suggest that the foundation of turbo machinery incorporating barrettes could be successfully utilized in places prone to seismic activity. Barrettes were demonstrated to protect the foundation by absorbing and diminishing the seismic loading, owing to their lateral resistance and substantial specific surface area. However, it was shown that barrettes alone were inadequate in mitigating vibrations caused by dynamic loading resulting from the machinery's rotational movement or seismic forces, particularly in poor soil conditions such as NEHRP D (clay soil). To overcome this limitation, the study proposed the utilization of geosynthetics in conjunction with barrettes, resulting in a significant reduction in vibrations on the upper deck in areas with unfavorable soil conditions. The soil was represented as interconnected elements with six freedom degrees (DOF), allocated to each raft mesh and individual barrettes nodes. The machine components were depicted as inflexible connections positioned on the upper surface of the foundation. The study examined a machine that was rotating at a speed of 3995 rpm, and it analyzed the imbalanced forces caused by the weight and rotational motion of the motor. Sine functions were integrated into the upper top deck to replicate the machine's harmonic loads that were transferred by rigid linkages. In addition, earthquake time history data were used at the raft base to enable seismic stimulation and subsequent vibration analysis.

Fattah et al. (2017) [6] conducted an experimental study on the performance and response of machine foundations situated on both dry and saturated sand. This study assessed a total of 84 models. The factors related to the footing were associated with the dimensions of the rectangular footing and its embedment depth. Two distinct sizes of rectangular steel model footings were evaluated, both at the surface and at a depth of 50 mm beneath the model surface. The study examined diverse soil conditions, including saturated and dry sand, at two relative densities of 80 % and 30 %. The footings' displacement amplitude was assessed with a vibration meter. The study's principal findings indicated that the maximum amplitude for foundations on dry sand models surpassed that of saturated sand by roughly 5.0–10 %. Furthermore, augmenting the footing size by a factor of two led to a decrease in the maximum displacement amplitude for both saturated and dry sand. Additionally, the ultimate foundation settlement (St) was noted to rise with an increase in saturation degree, dynamic force amplitude, and operating frequency. In contrast, it diminished with an increase in the relative density of sand, the modulus of elasticity, and the depth of embedding in the soil.

Bhattacharya (2019) [7], presented a practical example outlining the design process for a tabletop foundation supporting a 60 Hz steam turbine generator. This involved the creation of a finite element model using ANSYS software. The analysis results were then used to perform static design checks to ensure the foundation's stability under normal operating conditions. Additionally, dynamic analysis was conducted to assess the foundation's response to varying loads, paying particular attention to resonance effects and adherence to the amplitude limits specified by the machine manufacturer. Notably, this study did not account for seismic loads, focusing solely on conventional analysis and design procedures for the foundation's performance. In summary, Bhattacharya's work offered a step-by-step guide for the design of a tabletop foundation under typical operating conditions, utilizing finite element modeling and analysis to ensure its structural integrity and dynamic stability, albeit without considering seismic forces.

Kaream et al. (2020) [8], explored the impact of circular machine foundations on surface settlement, vertical displacement, and stress variations over cycles. They conducted experiments using a specialized setup on six laboratory model footings placed on separate beds of dense and medium dry sand. A 150 mm diameter steel model represented the footing, with dynamic load amplitudes of 0.25 tons and frequencies of 0.5, 1, and 2 Hz. Results showed that increasing frequency reduced settlement rate in both sand types, higher soil relative density reduced settlement, and increasing frequency and relative density decreased displacement amplitude.

Karmegam Rajkumaret et al. (2021) [9], conducted a study examining the impact of soil-structure interaction (SSI) on a torsional coupled turbo-generator (TG) machine foundation subjected to earthquake. They utilized a 3D finite element model for their analysis. The study also investigated the advantages of incorporating base isolators in TG foundations, taking SSI into account. Two eccentricity ratios for superstructure were considered to simulate torsional coupling, and the study focused on soft soil properties to analyze SSI effects. The findings of the study highlighted that neglecting the effects of torsional coupling could lead to alterations in natural frequencies and potentially result in unsafe designs. Additionally, the study revealed that increasing superstructure eccentricity led to higher deck accelerations and displacements. However, the incorporation of base isolators significantly reduced deck accelerations and displacements, demonstrating the advantages of using base isolators in protecting sensitive equipment from strong ground motion earthquakes. It's important to note that the effects of SSI, especially in soft soil conditions, led to reduced natural frequencies and increased participation of higher modes, amplifying the overall TG foundation response.

Abdulrasool et al. (2022) [10] conducted experiments involving rectangular and circular machine foundations situated on clay soil with variable saturation degrees, specifically 60 % and 100 %. Their primary objective was to assess the foundation's displacement amplitude under different operating frequencies. Additionally, they examined vertical displacements and stress within the soil beneath the foundation at three distinct points (0.5B, B, and 2B, where B represents the foundation width). Experimental outcomes revealed several key findings. Firstly, increasing the degree of soil saturation led to a significant reduction in foundation amplitude displacement, with a decrease of approximately 61 %. Furthermore, higher soil saturation levels resulted in a notable reduction in vertical stress, with a decrease of around 77 %. Importantly, alterations in the soil degree of saturation exerted a substantial effect on soil displacement, with the magnitude of this effect varying based on the depth of the point below the foundation.

This present work's novelty and main goal is to clarify the impact of some geometrical properties of frame foundations used for high-speed machine foundations like column length, column dimensions, and table-top slab thickness, on the frame foundation's natural frequencies and dynamic displacement response. These properties are thought to affect the performance of the frame structure and, consequently, the safety and performance of the turbo-machine during its operation lifetime.

## 2 ANSYS workbench program verification

**Fig. 1:**
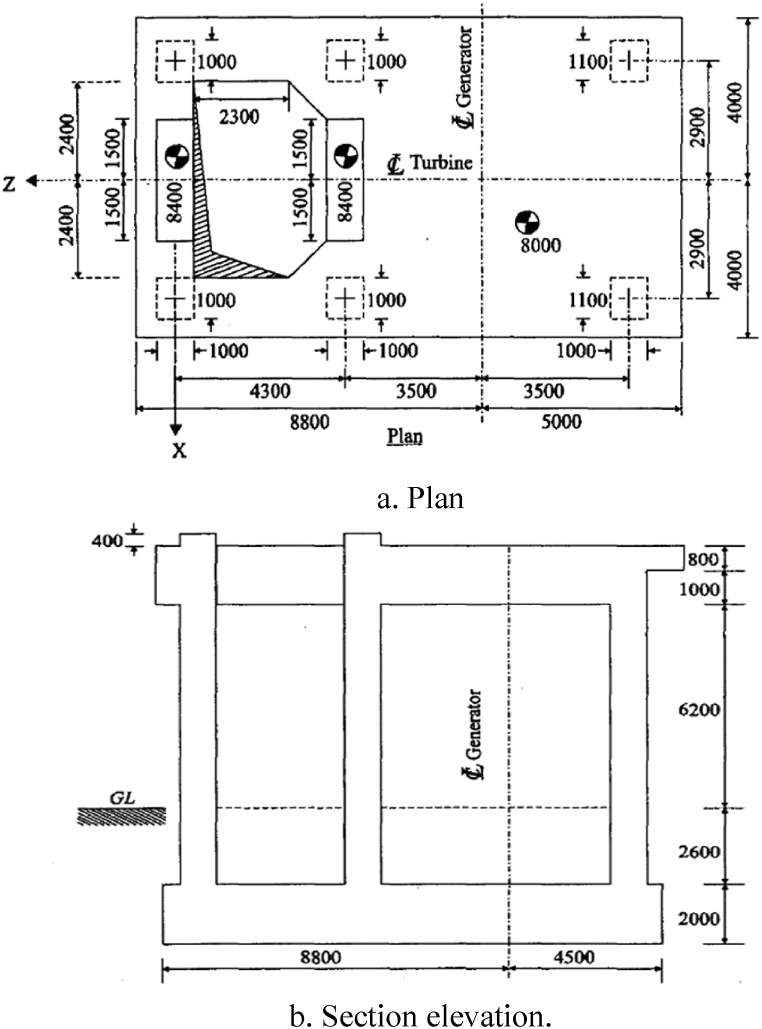
Bhatia's frame foundation details a) Plan b) Section elevation [11].

The ANSYS 21 Workbench was verified prior to the parametric study by comparing the results obtained from a finite element model representing a high-speed rotating machine's frame foundation. This model was sourced from Bhatia's renowned publication Foundations for Industrial Machines Handbook for Practicing Engineers [11]. The case study details, as shown in Fig. 1(a) and (b), were incorporated into ANSYS workbench 21 after constructing the frame in Ansys workbench and specifying the properties of the materials, as illustrated in Table 1. The boundary conditions of the frame were detailed in the sourcebook. Then, conducting a modal analysis to obtain the frame structure natural frequencies; two cases were adopted in calculating the foundation natural frequencies: the first one is the standard case where the machine mass is included, and the second one is done with the machine mass is excluded to account for neglecting the impact of the machine mass on the calculated natural frequencies. These natural frequencies are essential in the frame design to compare these frequencies with the rotating machine operating frequency; this step is important to ensure that the machine frequency does not match the system's natural frequencies, which will cause resonance and thereby affect the performance of the highly-cost machine.

**Table 1:** Physical properties of the concrete for finite element analysis of Bhatia (2008) [11].

**Properties of concrete**	**Value**
**Mass Density of Concrete ρ (kg/m**^**3**^**)**	2500
**Shear Modulus G (GN/m**^**2**^**)**	13
**Poisson's Ratio *μ***	0.15
**Young's Modulus E (GN/m**^**2**^**)**	30

The outcomes obtained from the modal analysis using ANSYS21 workbench for the case considering the mass of machine are tabulated in Table 2 for the primary 20 modes. The results of the FEA demonstrate a strong correlation with both dynamic and static outcomes results calculated by Bhatia (2008) demonstrating the precision of the current modeling. The results of the verification analysis are presented in a separate paper for the static and dynamic modal for the two cases [12]. It was concluded that the accuracy of the natural frequencies calculated was above 90 % if the machine mass was ignored.

**Table 2:** Modal analysis results for the work of Bhatia (2008) [11] and using ANSYS-21 [12].

**Mode No.**	**Freq.** **Hz (Bhatia)**	**Freq.** **Hz (Ansys)**	**Mode No.**	**Freq.** **Hz (Bhatia)**	**Freq.** **Hz (Ansys)**	**Mode No.**	**Freq.** **Hz (Bhatia)**	**Freq.** **Hz (Ansys)**	**Mode No.**	**Freq.** **Hz (Bhatia)**	**Freq.** **Hz (Ansys)**
1	2.95	2.9755	6	33.2	32.113	11	36.75	36.362	16	39.43	38.764
2	3.02	3.0458	7	35.57	32.304	12	36.78	36.849	17	39.83	39.518
3	3.67	4.0814	8	36.4	33.188	13	36.8	36.974	18	42.67	40.067
4	26.48	23.859	9	36.45	35.184	14	37.05	37.19	19	45.81	41.799
5	32.36	30.057	10	36.62	36.169	15	38.7	38.299	20	58.88	42.918

## 3 Problem description

**Fig. 2:**
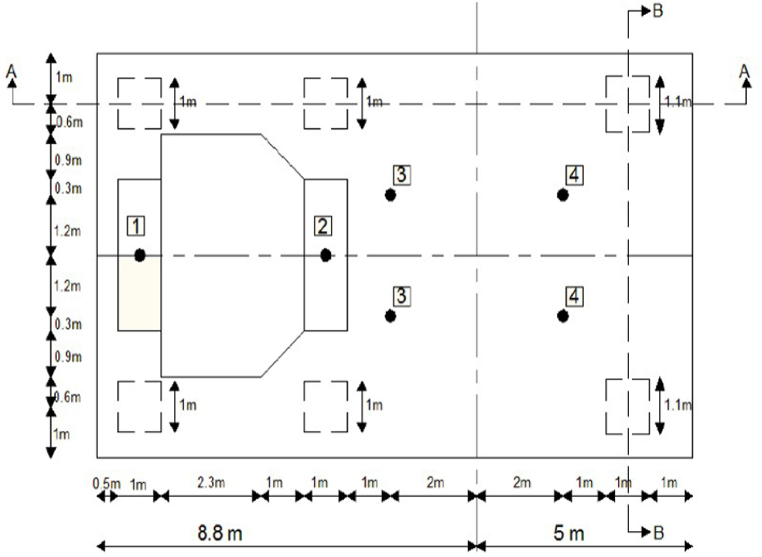
Tabletop dimensions and bearing locations of Bhatia's frame foundation [11].

The effect of the tabletop slab thickness, column length, and dimensions, or area, of a typical high-speed turbine frame foundation is investigated by using ANSYS Workbench 2021 R1 software. The same frame structure used for verification is adopted in this geometrical study. Table 3 presents the static and dynamic loading values generated by the rotor masses on the specified bearing locations of the frame foundation's tabletop. Fig. 2 displays the places of the load-bearing rotating parts, including the generator and turbine.

**Table 3:** Unbalance forces and machine loads at the top slab (Bhatia, 2008) [11].

Bearing locations	1	2	3	4	Total
Weight of Rotor (kN)	25	35	70	70	200
Weight of Machine (kN)	400	360	200	200	1160
Unbalance Forces					
Blade Loss Force (kN)	3	11	–	–	14
Longitudinal (kN)	2	3	6	6	17
Vertical/Lateral (kN)	5	7	15	15	42

### 3.1 Program data

**Fig. 3:**
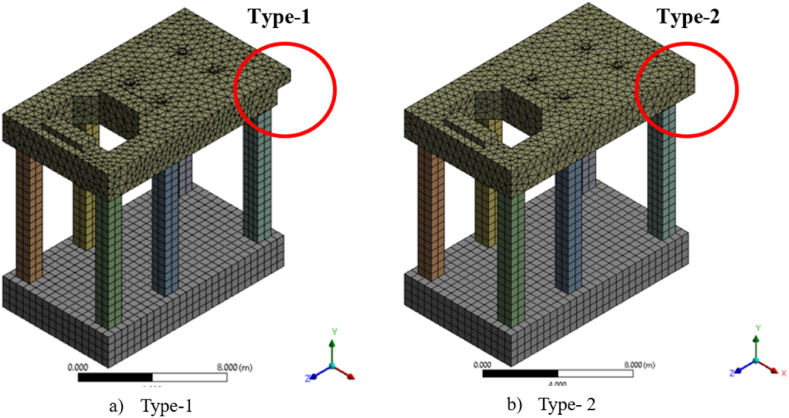
Finite elements meshing of the frame foundation a) type 1 b) type 2.

With specified dimensions and using Ansys Space Claim, the structure was initially drawn. The analysis of the 3-D frame structure is commenced using Ansys Workbench software. The frame foundation is modeled by using eight bodies that consist of a raft, six columns, and a tabletop. After assigning the properties of concrete material, the three-dimensional structure elements, consisting of three rotational and three translational, are meshed using element SOLID186, with a 500 mm element size, to have a regular shape and to increase the accuracy of the results for all raft and columns bodies elements while the table-top and due to the irregular shape was meshed by SOLID187 tetrahedron element. The meshed structure consists of around (32175–32681) nodes and (14557–14715) elements depending on the model type; type 1 is used to study column's length and area while type 2 is used to study slab thickness, as shown in Fig. 3.

The turbomachine masses load was distributed according to Table 3 by using a rigid behavior remote point masses to represent the rigid machine. This new technique is simpler and more accurate than the previous technique used by Bhatia and many authors, which modeled the masses of the machine using rigid links to apply the mass of the machine on the frame foundation at the machine's center of rotation. The total mass was applied to the specific bearing locations of the rotor machine on the frame, as mentioned in Table 3.

Boundary conditions were assigned to the structure by assigning zero displacement and rotation to vertical and horizontal directions of the bottom area of the raft, (Ux = Uy = Uz = θx = θy = θz = 0). The gravity load is applied to the structure and the results of the static analysis is used for the dynamic analyses as prestress forces.

### 3.2 Free vibration analysis

The first dynamic analysis is the free vibration modal analysis to determine the system mode shapes and the frequencies; “eigenvectors and Eigenvalues”. This analysis is performed by using Modal analysis using APDL solver incorporated in Ansys Workbench analysis systems. These mode frequencies are essential for comparison with the operating frequency of the turbo-machine and to certify that the system frequencies are well apart to overcome resonance possibility. Since this is a modal analysis, no load was applied during the analysis. No damping was given during the modal solution.

Modal analysis is performed with a maximum reached number of modes to be extracted of 400 and the range of (0–750) Hz to enhance the precision of the output and to ensure that the mass participation ratio will be more than 90 % in accordance with the different code's recommendations. Since the operating frequency of the machine is about 50 Hz, only the first 20 modes were adopted for comparison with Bhatia's (2008) results as mentioned in Table 2.

### 3.3 Steady state vibration analysis

The second type of dynamic analysis is the harmonic analysis. The same foundation with the identical meshed elements, after applying all dynamic forces, was examined, as detailed in Table 3, at designated bearing points in the vertical, horizontal rotational axis plane and in the machine's longitudinal axis. The analysis is conducted using harmonic response analysis utilizing a mechanical APDL solver integrated into Ansys Workbench 21 analytical systems. To replicate the actual loading scenario, horizontal and vertical dynamic forces were applied at designated locations with a 90-degree phase difference, as the two forces cannot act concurrently. The transient phase was disregarded because this is a harmonic analysis, and after a few rotational cycles, all forces arising from the transient phase will dissipate, leading to a steady state. A 5 % damping ratio was selected to prevent the structural response from becoming infinite at the resonant frequency during the analysis.

The harmonic analysis was conducted in two approaches, the first approach which is the recommended and faster approach with less time and data required for solving the problem, is conducted by using the mode superposition method in which the response of the structure and results are depending on of the modal analysis results. The mode shapes and frequencies were calculated for the principal mode shapes. The mass participating ratio was calculated and compared with the code's recommendations of 90 % to ensure the results were accurate, but since the lower raft face elements were fixed in translational and rotational degrees of freedom, preventing these elements from participating in the structure movement. The corresponding mass participating ratio was always below 90 percent even after increasing the required mode shapes to be extracted, which may put the results into question.

**Fig. 4:**
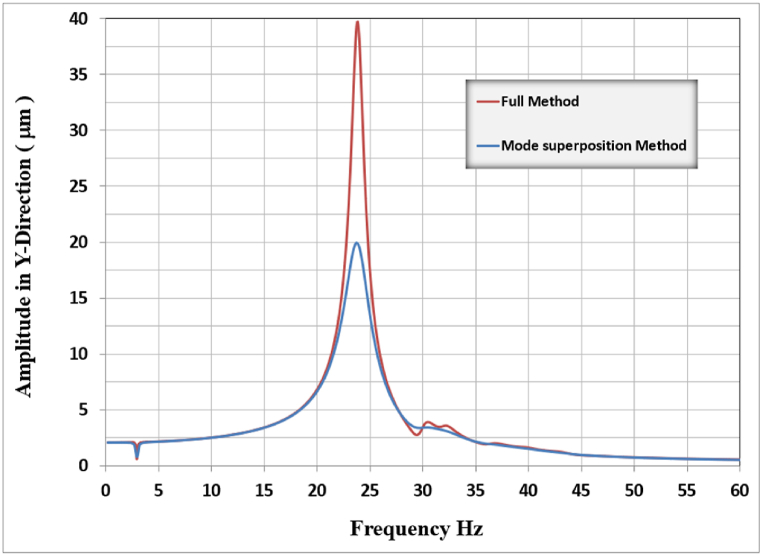
Full vs mode superposition method results for amplitude in y-direction.

The second approach, which is the direct full method, calculates the dynamic response of the system at each specified frequency. This method requires more time and data management but it guarantees accurate results. Since the mode shapes are necessary for analyzing and understanding the frame structure behavior, the two methods were used in the analysis but the full method results are used for calculating the system response and comparison since it is more accurate in results. Comparing the outcomes of the two approaches reveals that both methods yield a convergent vertical amplitude result when the frequencies are distant from the resonance frequency. However, as the frequencies approach the resonance zone, the full method produces an amplitude response approximately twice that of the mode superposition method, as illustrated in Fig. 4. Since the operating frequency of the turbo-machine is 50 Hz then the frequency limit to be accounted for as per codes is 60 Hz [13]. To calculate the response of the frame foundation, the adopted range of frequencies is used between (0-75) Hz with 0.25 Hz intervals between them which gives 300 points of the calculated response. This is much higher than the recommendations of a 2 Hz interval but seeking for more accurate results is adopted during this study. In addition to that, user-defined points of closely spaced intervals are used to capture precisely the curvature of the response near resonance at each of three directions. This method led to an increase in the time of analysis but the more accurate responses were captured.

## 4 Scope of the study

This study aims to investigate several parameters of the frame foundation components, such as the effect of top deck thickness, column length, column area, or dimensions, since these parameters may affect the total static and dynamic response and the cost of the system. The thickness of the top deck was investigated using five thicknesses of (1.0, 1.2, 1.4, 1.6 and 1.8) m. Bhatia (2008) stated that the weight ratio of the table-top slab used for the frame foundation is very high, but suppliers determine the thickness. Hence, this situation will be studied from a dynamic point of view. The effect of the thickness is compared for the five different cases to clarify the effect of the table-top slab thickness on the natural frequencies of the frame foundation. In addition to that, the effect on the frame responses to the harmonic loading was also examined.

The column's effect on the performance of the frame foundation is separated into two components; the first one is the length of the column since the column length may control the behavior of the column as a short or slender column, which affects the static and dynamic behavior. No recorded information on how this will dynamically affect the foundation system's response. The following column lengths of (6.8, 7.8, 8.8, 9.8, and 10.8) m are used in this study. The second component is the change in area or column dimensions, which also affects the static design, but again, no information is recorded about its influence on the dynamic response. The columns are assumed to be square in cross-section with horizontal dimensions of (0.8, 0.9, 1.0, 1.1, and 1.2) m, which corresponds to the column's area of (0.64, 0.81, 1.0, 1.21, and 1.44) m^2^.

## 5 Results and discussion

The frame foundation's static analysis is conducted to verify the results with Bhatia's works in his aforementioned book. The modal analysis was conducted for many scenarios, encompassing varying slab thicknesses, column lengths, and dimensions. The modal analysis employed the static foundation to determine the natural frequencies, disregarding any loading types aside from implementing fixed boundary conditions at the base of the raft foundation. The identical meshed frame performed harmonic analysis following the application of all dynamic masses and loads associated with various components of the turbine generator equipment.

**Fig. 5:**
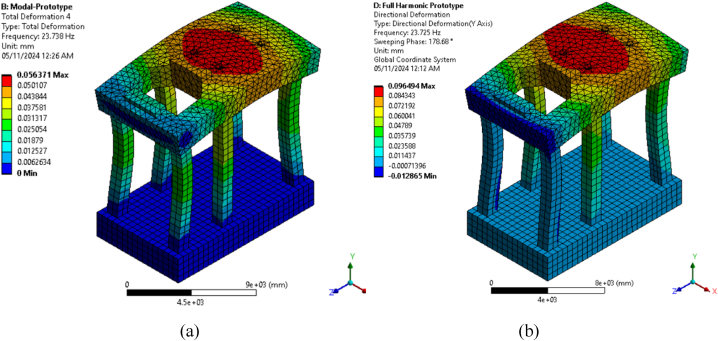
Contour of vertical deformation for prototype model type-2 for a) modal analysis and b) harmonic analysis at maximum frequency.

All dynamic loads were applied simultaneously in all directions in the harmonic analysis, incorporating real phase differences among in-plane, vertical, and lateral stress. Bhatia employed the loading of each direction individually in a single run; however, in practice, all loads are applied in all directions at designated locations and times. This methodology is utilized during harmonic analysis for all cases. The results of the full method are shown again, as they are more precise. Fig. 5 illustrates the deformation contours for the first vertical mode shape, mode number 4, derived from modal Fig. 5(a) and harmonic analyses Fig. 5(b) at the maximum frequency of (23.725) Hz.

### 5.1 Effect of table-top thickness

To investigate the effect of table-top slab thickness, the column's cross-section area of [1] m^2^ and lengths of (8.8) m were fixed for all cases; these values are based on the prototype dimensions. The remote masses point elevations were varied so that in all cases, the height of mass points was fixed to the same elevation above the table-top slab upper surface.

**Fig. 6:**
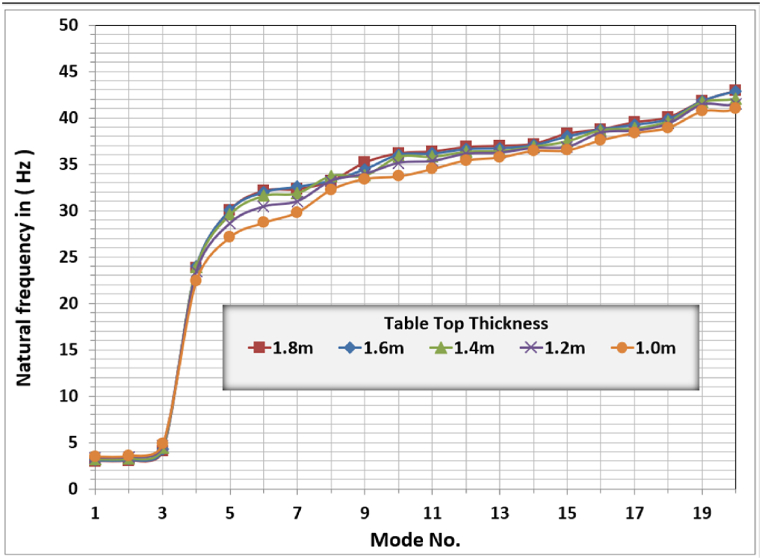
Natural frequencies for different table-top slab thicknesses.

Fig. 6 shows the modal analysis results for different slab thicknesses. The general behavior for all modes is the same, and the change in slab thickness did not change the system's behavior. The translational modes are the first and second modes; the torsional mode is the third mode, and the fourth mode is the vertical mode, which is the most interesting mode from the designer's point of view.

Fig. 6 shows that in the studied cases, the slab thickness has a marginal effect on the natural frequencies of the foundation. This may be because all used thicknesses represent rigid slabs in behavior, and any increase in slab mass is accompanied by an increase in slab stiffness. Their combined effect has a marginal effect on the natural frequencies or modal behavior.

**Fig. 7:**
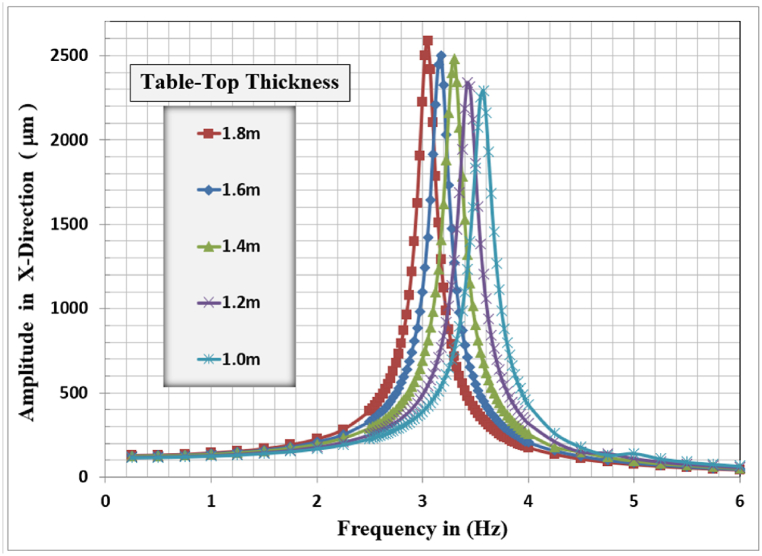
Displacement amplitude at resonance for different slab thicknesses in X-direction.

**Fig. 8:**
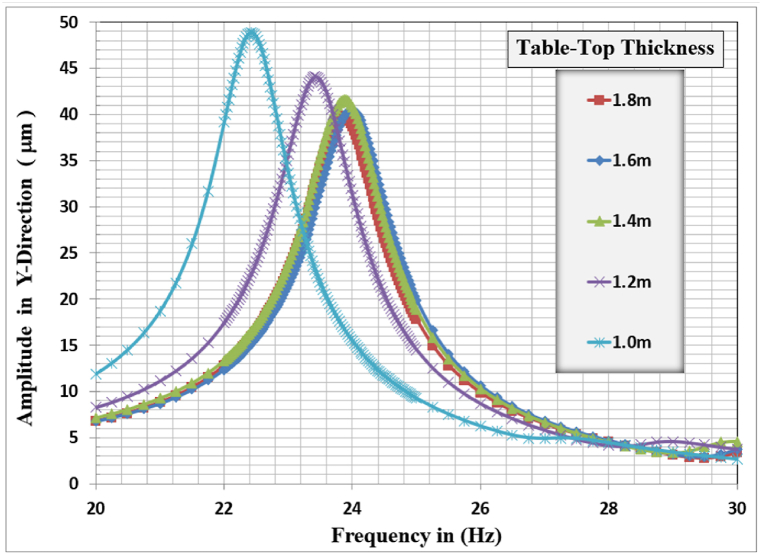
Displacement amplitude at resonance for different slab thicknesses in Y-direction.

**Fig. 9:**
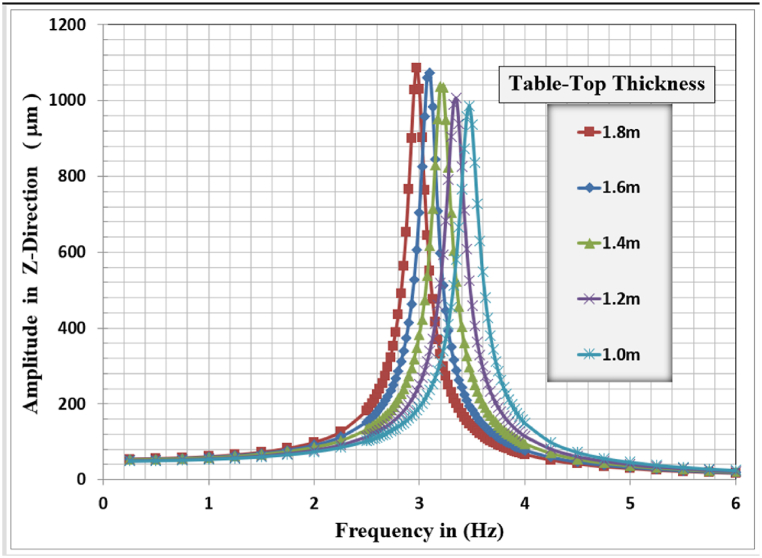
Displacement amplitude at resonance for different slab thicknesses in Z-direction.

Fig. 7, Fig. 8, Fig. 9 show the results of displacement amplitude response in X, Y, and Z directions for harmonic analysis for many slab thicknesses near the resonance frequency of the system in each specified direction. The displacement response is higher for the horizontal translational modes in X and Z directions because the frame structure is laterally unrestrained in its top locations.

In X-Direction, the higher displacement response of 2586.9 μm, corresponding to a resonant frequency of 3.05 Hz, was observed for the slab thickness of 1.8 m and the lower response of 2292.3 μm, corresponding to a resonant frequency of 3.575 Hz, is for the 1.0 m slab thickness. The general behavior is that with increasing slab thickness, the resonance frequency for the frame foundation shifted to the left, and decreased, but with a higher corresponding lateral displacement response.

In the Y-direction, which is the most interesting direction from the designer's point of view, the higher displacement response of 48.875 μm, corresponding to a resonant frequency of 22.425 Hz, was observed for the slab thickness of 1.0 m and the lower response of 39.739 μm, corresponding to a resonant frequency of 23.85 Hz, is for the 1.8 m slab thickness. The general behavior is that with increasing the slab thickness, the vertical displacement response decreases marginally. The resonance frequency for the frame foundation at which maximum response is calculated for the different slab thicknesses did not show a consistent behavior but they were all between 22.4 and 23.85 Hz.

In Z-Direction, the higher displacement response of 1086.0 μm, corresponding to a resonant frequency of 2.975 Hz, was observed for the slab thickness of 1.8 m and the lower response of 985.16 μm, corresponding to a resonant frequency of 3.475 Hz, is observed for the 1.0 m slab thickness. The general behavior is that with increasing slab thickness, the resonance frequency for the frame foundation shifted to the left, and decreased, but with a higher corresponding lateral displacement response.

**Fig. 10:**
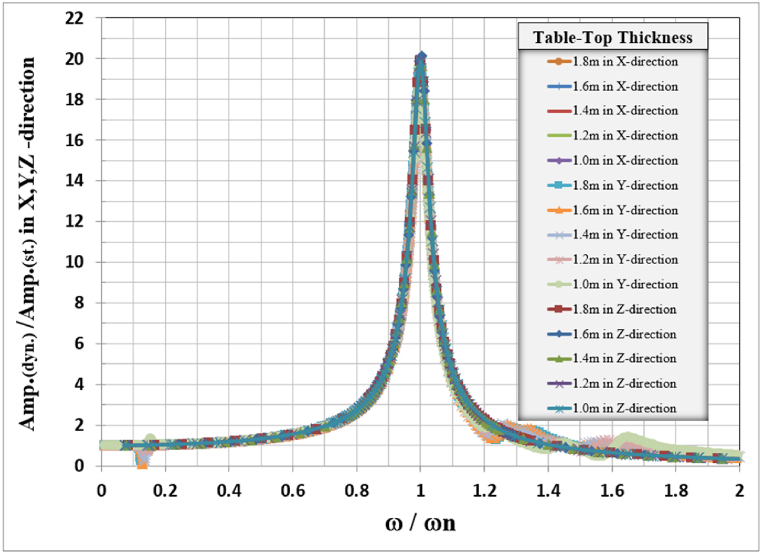
Normalized displacement amplitude and frequencies for X, Y, and Z-direction for different table-top slab thicknesses.

In all vertical and lateral cases, the resonance frequency was away from the turbomachine operating frequency of 50 Hz. The general responses for the analyzed foundation in three directions, X, Y, and Z, are normalized by dividing the frequencies used to calculate the response in any direction by the natural frequency of the frame for the same direction with corresponding table-top slab thickness as shown in Table 4, and also the amplitude of the responses was normalized by dividing it by the static displacement i.e. when the loading frequency is zero, the total normalized responses are shown in fig. (10).

**Table 4:** Natural frequencies for different tabletop slab thicknesses.

Mode No.	Tabletop slab thickness in (m)				
	1.8	1.6	1.4	1.2	1.0
	Natural frequency for frame (Hz)				
1	2.9755	3.0903	3.2124	3.3419	3.4713
2	3.0458	3.1682	3.2967	3.4344	3.5682
4	23.859	23.987	23.886	23.436	22.429

### 5.2 Column's area effect

To investigate the effect of the column's cross-sectional area, column's length of 8.8 m was fixed for all cases, and other frame dimensions were based on the prototype dimensions. The remote mass points were, in all cases, fixed at the same elevation. The columns are assumed to be square in cross-section with horizontal dimensions of (0.8, 0.9, 1.0, 1.1, and 1.2) m, which corresponds to the column's area of (0.64, 0.81, 1.0, 1.21 and 1.44) m^2^. Thus, for the 0.8 column's dimension, four columns are (0.8∗0.8) m and two columns of (0.8∗0.9) m, near the generator to maintain the same shape for the prototype columns for comparison purposes.

**Fig. 11:**
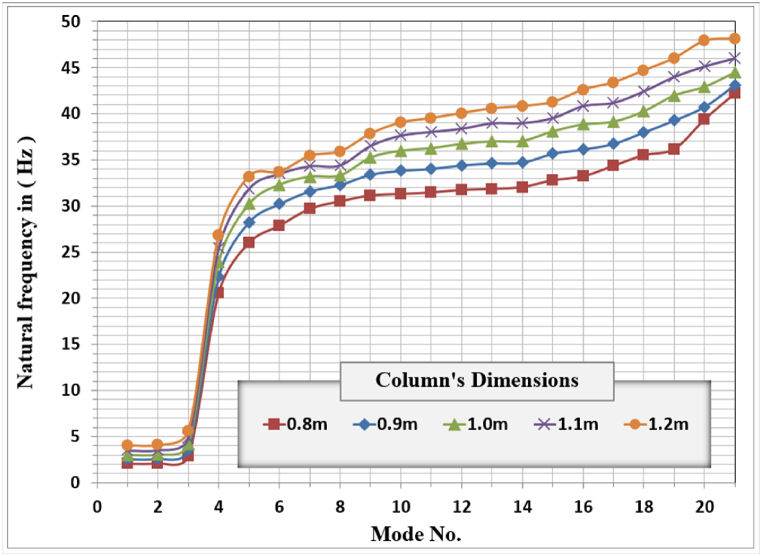
Frame foundation's Natural frequencies for different column dimensions.

Fig. 11 shows the modal analysis results for different column areas or dimensions. The behavior for all modes is the same, and changing the column area or dimensions did not change the system's mode number sequence. Translational modes are the first and second modes, the torsional mode is the third mode, and the vertical mode is the fourth mode.

The effect of the column's area is remarkable on the natural frequency of the frame foundation. For any mode number, the increasing of the column's dimension will increase the corresponding natural frequency to cause resonance in the system and vice-versa. The natural frequency for the first vertical mode shape is 20.57 Hz for the 0.8 m column's dimension while for the 1.2 m, the corresponding natural frequency is 26.846 Hz. This increase in the natural frequency is mainly due to the increase in the system stiffness.

**Fig. 12:**
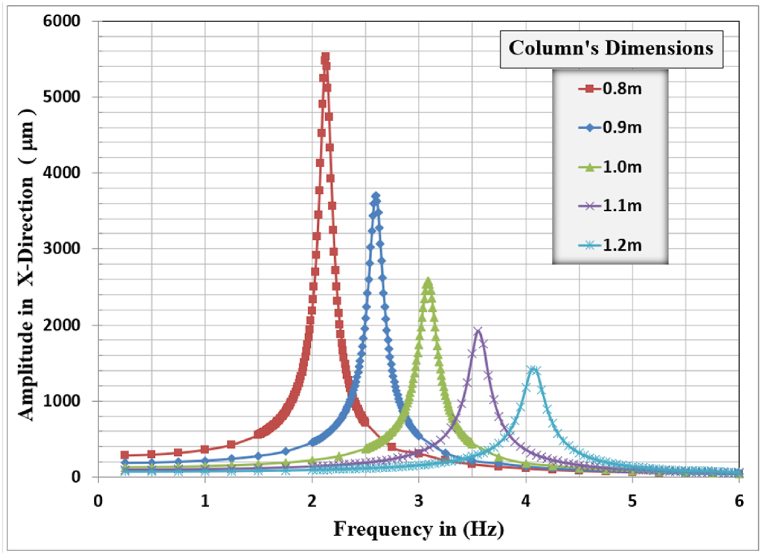
Amplitude displacement at resonance for x-direction for different column dimensions.

**Fig. 13:**
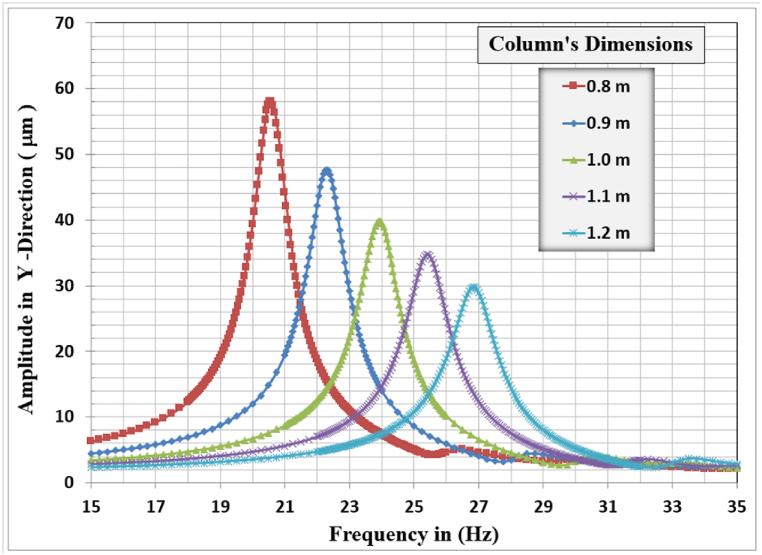
Amplitude displacement at resonance for y-direction for different column's dimensions.

**Fig. 14:**
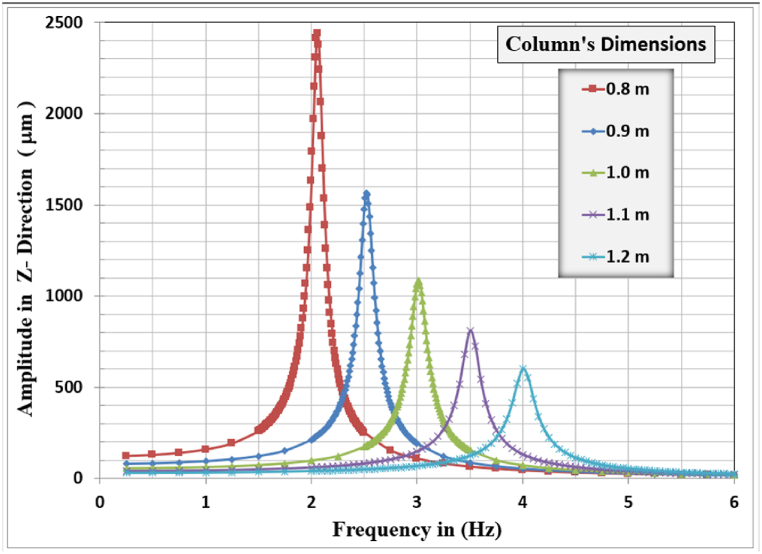
Amplitude displacement at resonance for z-direction for different column dimensions.

Fig. 12, Fig. 13, Fig. 14 show the results of displacement amplitude response in X, Y, and Z directions for harmonic analysis for many columns’ dimensions near the resonance frequency of the system in each specified direction. The displacement response is higher for the horizontal translational modes in the X and Z directions because the frame structures are laterally unrestrained in their top locations.

In the X-direction, the higher displacement response of 5533.5 μm, corresponding to a resonant frequency of 2.13 Hz, is observed for the column's dimensions of 0.8 m, and the lower response of 1408.0 μm, corresponding to a resonant frequency of 4.05 Hz, is observed for the 1.2 m column's dimensions. The general behavior is that with increasing the column's dimensions, the resonance frequency for the frame foundation shifted to the right, increasing, but with a lower corresponding lateral displacement response.

In the Y-direction, the higher displacement response of 58.068 μm, corresponding to a resonant frequency of 20.55 Hz, was observed for the column's dimensions of 0.8 m and the lower response of 29.764 μm, corresponding to a resonant frequency of 26.85 Hz, is observed for the 1.2 m column's dimensions. The general behavior is that with increasing the column's dimensions the vertical displacement amplitude decreases remarkably, also the resonance frequency for the frame foundation at which maximum response is calculated for the different column's dimensions increases with the increase of the column's dimensions.

In the Z-direction, the higher displacement response of 2439.7 μm, corresponding to a resonant frequency of 2.06 Hz, is observed for the column's dimensions of 0.8 m and the lower response of 599.31 μm, corresponding to a resonant frequency of 4.0 Hz, is observed for the 1.2 m column's dimensions. The general behavior is that with increasing the column's dimensions, the resonance frequency for the frame foundation shifted to the right, increasing but with a lower corresponding lateral displacement response.

In all vertical and lateral cases, the resonance frequency was away from the turbo machine operating frequency of 50 Hz.

**Fig. 15:**
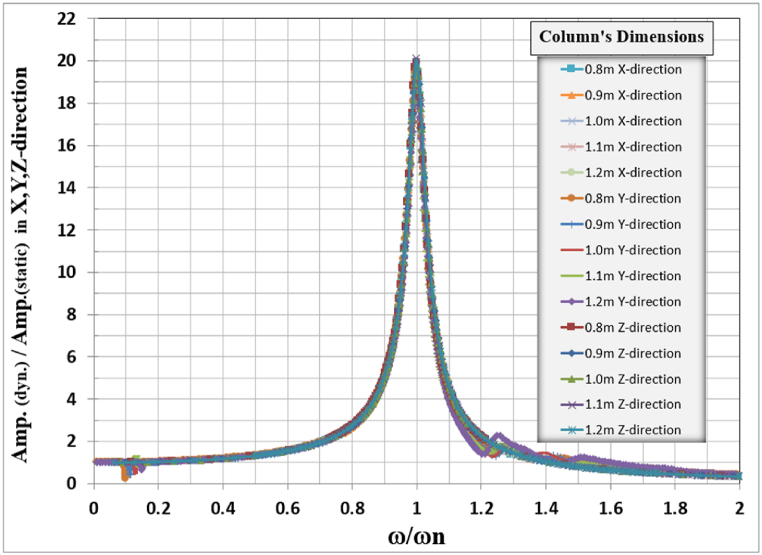
Normalized displacement amplitude and frequencies for X, Y, and Z-direction for different column dimensions.

The general responses for the analyzed frame in three directions X, Y, and Z are normalized by dividing the frequencies used to calculate the response in any direction by the natural frequency of the frame for that direction with the corresponding column's dimension as shown in Table 5. Also, the response amplitude was normalized by dividing it by the static displacement i.e. when the loading frequency is zero, the total normalized responses are shown in Fig. 15.

**Table 5:** Natural frequencies for different column dimensions.

Mode No.	Column's dimension (m)				
	0.8	0.9	1.0	1.1	1.2
	Natural frequency for frame (Hz)				
1	2.0578	2.5224	3.0128	3.5072	4.0071
2	2.128	2.5963	3.0856	3.5589	4.0733
4	20.57	22.301	23.923	25.425	26.846

### 5.3 Column's length effect

To investigate the effect of the column's length, the prototype slab, raft, and column's dimensions are adopted for all cases, and all loads and remote masses are applied at specified bearing locations above the table-top slab upper surface. The columns are assumed to be square in shape; four columns are (1.0x1.0) m, and two columns are (1.0x1.1) m, near the generator side. To investigate the column length effect on the displacement response of the frame, the following lengths (6.8, 7.8, 8.8, 9.8, and 10.8) m are used.

**Fig. 16:**
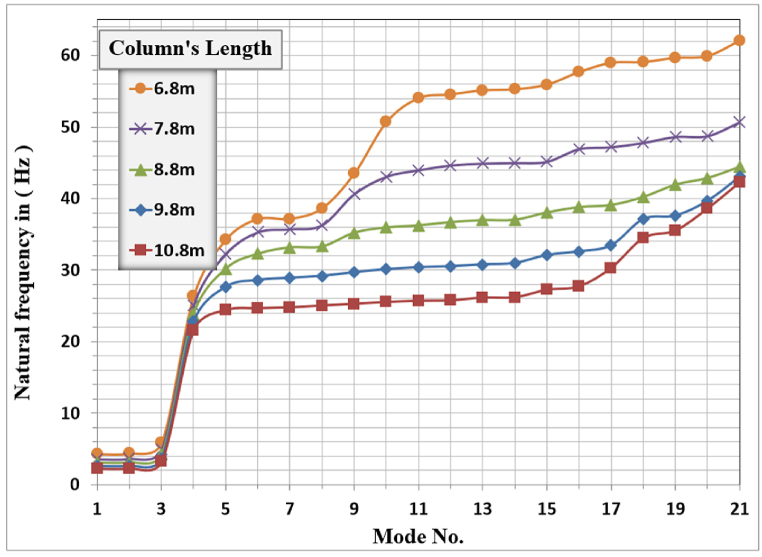
Natural frequencies of the foundation for different Column lengths.

Fig. 16 shows the modal analysis results for different column lengths. The behavior is not the same for all modes, and changing the column length did change the system's mode number sequence. Nevertheless, the translational modes are the first and second modes, the third mode is torsional, and the vertical mode is the fourth mode.

The column's length effect is remarkable on the modal behavior of the system more than that observed for the column's dimensions, it's observed that for the same mode number of the frame foundation. The increase in the column's length will decrease the corresponding natural frequency required to resonate the system and vice-versa. The natural frequency for the first vertical mode shape “mode 4” is 21.614 Hz for the 10.8 m column's length while for the 6.8 m column's length, the natural frequency is 26.347 Hz. The reduction in natural frequencies is due to increasing the column's length and the slenderness ratio of the column thus decreasing the stability and frame structure stiffness. For the prototype model, the column's length is 8.8 m, and the corresponding natural frequency is 23.932 Hz, this value is somewhat different from the 23.859 Hz tabulated in Table 2 because the frame shape used here Concluded the cantilever parts in the analysis.

**Fig. 17:**
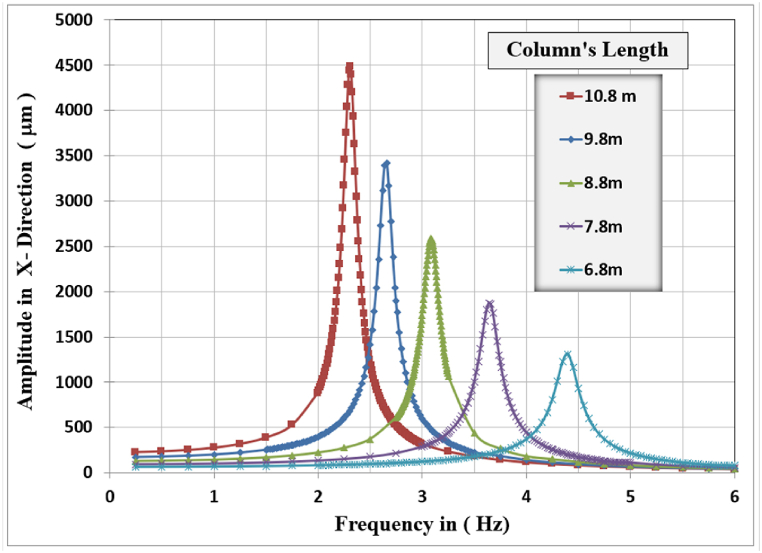
Amplitude displacement at resonance in the x-direction for different column lengths.

**Fig. 18:**
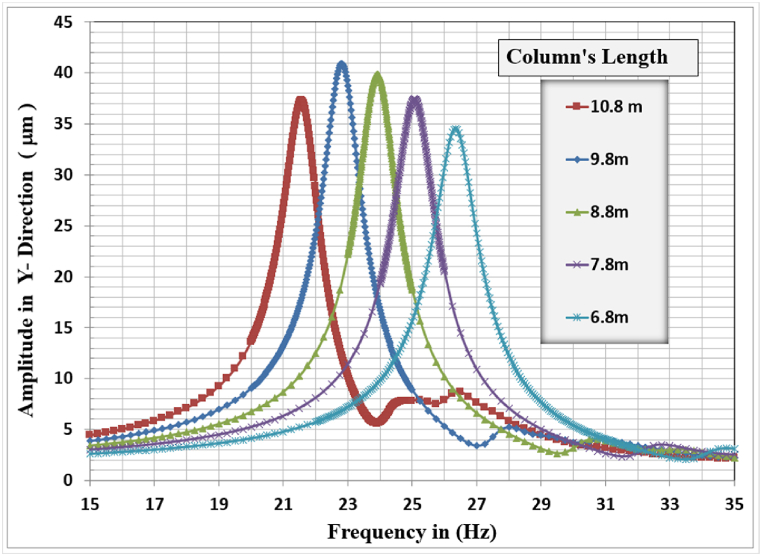
Amplitude displacement at resonance in y-direction for different column lengths.

**Fig. 19:**
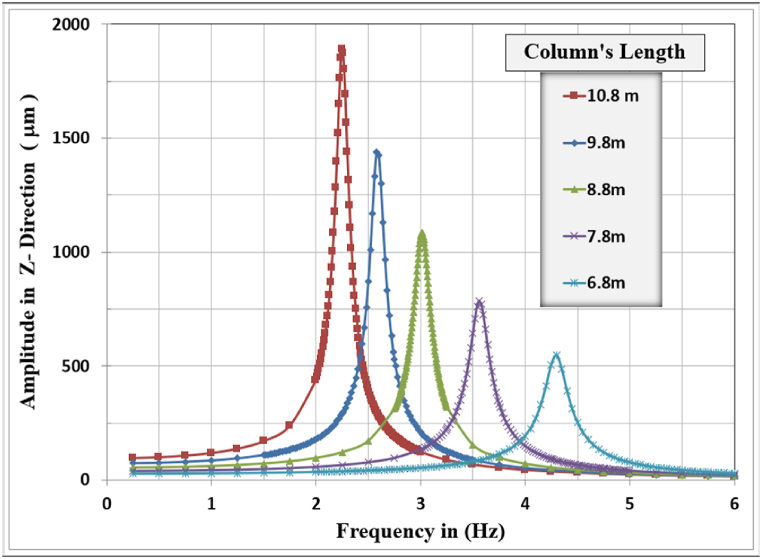
Amplitude of displacement at resonance in z-direction for different column lengths.

Fig. 17, Fig. 18, Fig. 19 show the results of displacement amplitude for harmonic analysis for different column lengths in the X, Y, and Z directions respectively. The displacement response is higher for the horizontal translational modes in X and Z directions than the vertical amplitude, because of increasing the slenderness ratio with increasing the length of columns.

In the X-direction, the higher response of 4486.7 μm at a frequency of (2.31) Hz for the (10.8) m column length while decreasing the column's length to (6.8) m produces a displacement of (1312.1) micrometer at a frequency of (4.4) Hz which is the lower calculated amplitude in the X-Direction.

In Y-Direction, the behavior is not clear, the amplitude shows convergent values for all the selected lengths except for the 10.8 m length which shows less amplitude than other lengths. The higher response of 40.928 μm at a frequency of 22.8 Hz for the 9.8 m column length. The lower value of maximum amplitude calculated was 34.542 μm observed at a frequency of 26.35 Hz for the length of 6.8 m which is the minimal calculated maximum amplitude in the Y-direction between all cases. The effect of the length of columns is not dominant for the y-direction amplitude and could be considered insignificant on the vertical displacement response. The increasing column length decreases the required frequency to cause resonance.

In the Z-direction, which is similar in behavior to the x-direction, the higher response of 1891.8 μm at a frequency of (2.25) Hz for the (10.8) m column length while decreasing the column's length to (6.8) m produces a displacement of (546.64) micrometer at a frequency of (4.3) Hz which is the lower calculated amplitude in the X-Direction.

**Fig. 20:**
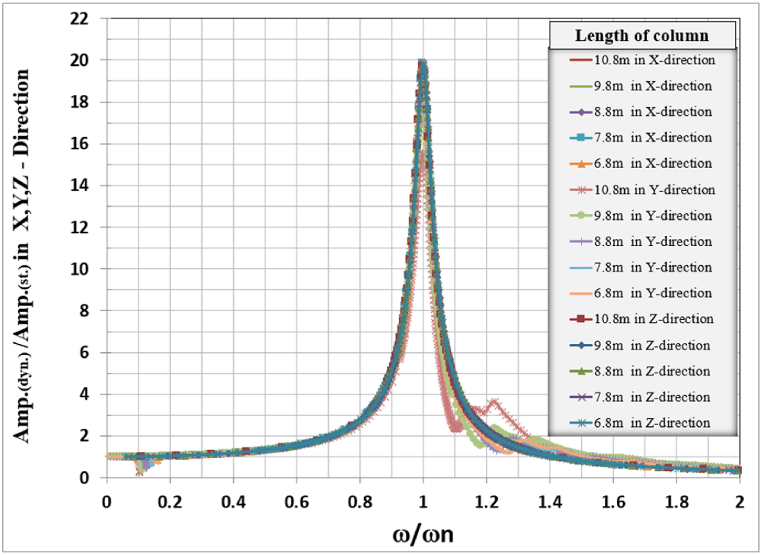
Normalized displacement amplitude and frequencies for X, Y, and Z-direction for different column length.

The general responses for the analyzed frame in the three directions X, Y, and Z are normalized by dividing the frequencies used to calculate the response in any direction by the frame's natural frequency for that direction with the corresponding column's length as shown in Table 6. Also, the response amplitude was normalized by dividing it by the static displacement i.e. when the loading frequency is approaching zero, the total normalized responses are shown in Fig. 20.

**Table 6:** Frame natural frequencies for different column lengths.

Mode No.	Column's length (m)				
	10.8	9.8	8.8	7.8	6.8
	Frame natural frequency (Hz)				
1	2.252	2.5879	3.0128	3.5627	4.2947
2	2.3083	2.6517	3.0856	3.6466	4.3924
4	21.614	22.819	23.923	25.076	26.347

**Fig. 21:**
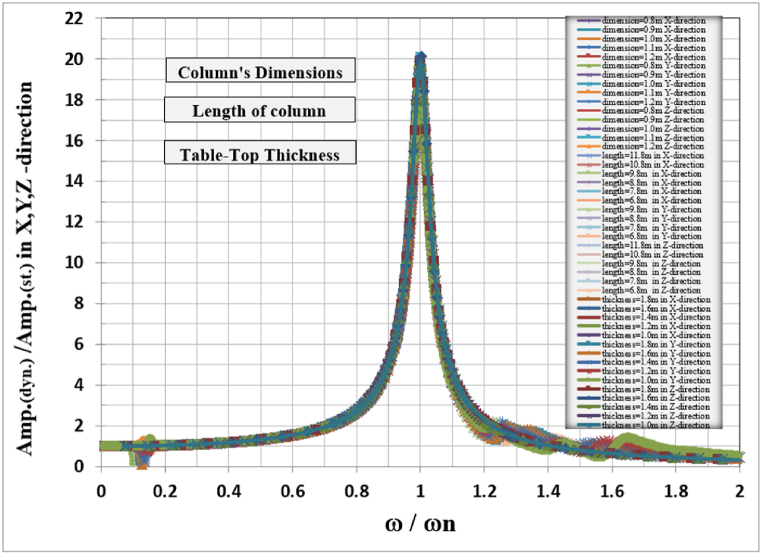
Normalized displacement amplitude and frequencies for X, Y, and Z-direction for different table top thicknesses, column dimensions, and column length.

The normalized behavior of each frame foundation slab and column dimension and length are identical in behavior in the three principal directions.

The total combined effects of table top thickness column dimension and column length for X, Y, and Z-direction are combined in one figure for each direction as shown in Fig. 20, it is clear that there is a unique curve for frame foundation relating the normalized frequency vs normalized amplitude for the studied case when damping is taken as 0.05 %.

The dynamic study of turbine foundations requires meticulous attention to detail in modeling and result interpretation (see Fig. 21). Jayarajan and Kouzer [14] emphasized several concerns with the mathematical modeling of structures, machinery, and soil for the dynamic study of foundation systems. The finite element approach is an effective instrument for modeling and dynamically analyzing turbo-generator foundations.

On the other hand, Rajkumar and Ayothiraman [9] concluded that the impact of SSI causes a decrease in the inherent frequencies of the turbo-generator foundation when it is placed on soft soil conditions. Additionally, SSI leads to an increase in the participation of higher modes, which amplifies the reaction.

Gaul [15] concluded that the impact of shear stresses at the boundary between soil and base is limited by the assumptions of completely smooth and completely welded contact. The overall system reaction is assessed by integrating the soil and foundation structures through a substructure technique.

## 6 Conclusions

All the results obtained in this study are from finite element analysis for the selected material properties, loading type, and boundary conditions. So, the results are limited to these conditions. The following conclusions could be obtained.

1. Increasing the thickness of the frame foundation tabletop slab has a marginal effect on the natural frequencies and the frame's dynamic responses during modal and harmonic analysis.
2. Increasing the column area has a remarkable effect on increasing the frame foundation's natural frequencies and decreasing the dynamic vertical and lateral displacement amplitude of the frame structure during harmonic analysis.
3. Increasing the column's length has a remarkable effect on decreasing the frame foundation's natural frequencies and increasing the frame structure's dynamic lateral displacement amplitude during harmonic analysis. Still, the effect is marginal for the vertical response.
4. There is a unique normalized frequency vs normalized amplitude response curve for frame foundation in X, Y, and Z direction.

## CRediT authorship contribution statement

**Ameer A. Ahmed:** Methodology, Investigation. **Mohammed Y. Fattah:** Validation, Supervision. **Makki K. Mohsen:** Writing – original draft, Validation, Supervision.

## Data availability statement

Most datasets generated and analyzed in this study are comprised in this submitted manuscript. The other datasets are available on reasonable request from the corresponding author with the attached information.

## Declaration of competing interest

The authors have no conflict of interest.
